# Co-Regulation as a Support for Older Youth in the Context of Foster Care: a Scoping Review of the Literature

**DOI:** 10.1007/s11121-023-01531-3

**Published:** 2023-04-21

**Authors:** Desiree W. Murray, Hannah Rackers, Aleta Meyer, Kelly Jedd McKenzie, Karin Malm, Kristin Sepulveda, Catherine Heath

**Affiliations:** 1 Center for Health Promotion and Disease Prevention, University of North Carolina, 1700 Martin Luther King Blvd, Campus, Box 7426, Chapel Hill, 27599 USA; 2 Youth Development and Child Welfare, Child Trends, Bethesda, MD 20814 USA; 3 Office of Planning, Research, and Evaluation, Administration for Children and Families, Washington DC, 20201 USA; 4 Children’s Bureau, Administration for Children and Families, Washington DC, 20201 USA

**Keywords:** Co-regulation, Foster care, Youth

## Abstract

Co-regulation is a relatively new theoretical framework for interventions that connects developmental science to adolescent needs and provides strategies that can be applied across contexts. It also has value in shifting the focus of interventions to the role of relationships and interactions with caring adults, as well as supportive environments. This framework may be particularly salient for older youth with foster care experience whose relationships with adults and availability of developmental supports are disrupted. To understand how co-regulation aligns with current understanding of needs and supports for this population, we conducted a scoping review that involved systematically searching four databases, coding and charting relevant information, and actively engaging expert consultants and other stakeholders. Across 46 primarily descriptive articles, co-regulation was discussed most often in relation to relationships, as expected (89% of articles). Despite theoretical and empirical evidence of the benefits of supportive environments and intentional day-to-day interactions in promoting developmental skills and competencies, these two domains of co-regulation were referenced much less (39% and 28%, respectively). Results highlight opportunities for co-regulation supports that can be provided to older youth with foster care experience by caring adults and near-aged peers in a wide range of roles. Notable limitations in the literature were identified in applying co-regulation within the context of employment and career readiness, healthy relationships, and teen parenting. Also under-researched is the role of adult self-regulation skills and co-regulation approaches for youth from diverse backgrounds, including those who identify as LGBTQ or have disabilities. Considerations for practice and future research are provided.

**Supplementary Information:**

The online version contains supplementary material available at 10.1007/s11121-023-01531-3.

Many youth in foster care experience stress and trauma before and during involvement in the child welfare system, increasing their need for social-emotional supports. In particular, they often face chronic stress related to poverty and a myriad of related stressors like food insecurity and inconsistent or unsafe living situations (Barth & Lloyd, 2010). They are at high risk for complex trauma (Greeson et al., 2011; Salazar et al., 2013), which can disrupt the stress management system and contribute to psychiatric difficulties (Greeson et al., 2011; Salazar et al., 2013). Chronic and/or overwhelming stress also interferes with development of self-regulation (i.e., managing thoughts and feelings to achieve goals, solve problems, and control impulses; Murray et al., 2019), which is foundational for lifelong well-being across a range of domains from mental health and emotional well-being to physical health, academic achievement, and socioeconomic success (Moffitt et al., 2011; Shonkoff et al., 2012).

Unfortunately, the US child welfare system presents challenges to effectively addressing these needs due to a variety of factors, such as a workforce with high turnover rates and large caseloads, changing policies and shifting responsibilities, and difficulties recruiting and retaining foster parents for older youth (Child Welfare Information Gateway, 2016; NRCDR, 2015a). For youth removed from their homes, foster care placement disrupts their social networks and opportunities for normalcy (AECF, 2015). Placement instability and congregate care further decrease access to consistent caregivers (Children’s Bureau, 2020). And as with many public systems, systemic racism, implicit biases, and lack of cultural competency among professionals can interfere with supportive relationships between older youth and adults (Dettlaff, 2015).

## Limitations of Current Social-emotional Supports for Older Youth

Although it is clear that consistent and caring relationships are critical for social-emotional development and long-term well-being for all adolescents, intervention research with this age group is much more limited than for younger children (NASEM, 2019). For example, behavior management strategies implemented in the context of warm, responsive relationships are well-established for promoting self-regulation for younger children (Frosch et al., 2021; Leijten et al., 2022). However, for older youth, specific practices for promoting well-being and resilience in the context of stress are much less well-defined and validated. To ensure that intervention approaches are developmentally informed, there should be consideration of adolescents’ needs to build a sense of identity, autonomy, and agency, as well as the importance of peers. Additionally, current neuroscience with adolescents suggests specific opportunities for intervention, although translational work is lacking (Dahl et al., 2018).

Application of developmental science suggests that caring adults should gradually shift power in their relationships with youth as they get older, which increases opportunities for youth to make and learn from their decisions while at the same time protecting them from high-risk situations (e.g., monitoring and setting boundaries) and teaching lifelong skills necessary for intimate relationships, managing difficult emotions and stress, and resolving conflict (Meschke et al., 2012; Murray et al., 2019). Family connections and adult guidance also remain important for young adults, especially with complex decision-making and challenging situations. Unfortunately, the literature provides limited guidance on specific practices that caring adults can use to address these developmental needs, and conceptual models for providing these supports are lacking (Meschke et al., 2012).

For older youth in foster care, approaches for supporting their social-emotional development are more complex given placement disruptions and service system policies (NASEM, 2019). Some evidence suggests that responsive and nurturing relationships with foster care parents and kinship caregivers, along with coaching and other skill-building opportunities, may be effective in reducing behavior problems (Kemmis-Riggs et al., 2018), although evidence-based programs do not appear widely used and foster caregivers identify need for additional guidance for managing day to day challenges (Benesh & Cui, 2017; Hebert & Kulkin, 2018). When youth in foster care are asked to share their perspectives, they describe a need not only for connection and belonging, but also for structure, boundaries, and guidance for the future (Storer et al., 2014). For youth in institutional care, there is a need not only for meaningful relationships with adults, but also for emotional safety (Fathallah & Sullivan, 2021).

Individual therapy is also used to promote the mental health of youth in foster care, particularly when they exhibit challenging behaviors. However, mental health services are considered difficult to implement for this population, and their effectiveness is unclear (Hambrick et al., 2016). Mental health approaches also focus primarily on changing the behaviors of the youth, overlooking the importance of the social-ecological environment, including policies and cultural practices (Biglan, 2018).

Finally, mentoring has been used to promote positive youth development by building relationships between youth and non-parental adults (Dubois & Karcher, 2013). However, one-on-one mentoring with youth often lacks intentionality, with a majority of mentored youth reporting that these relationships do not promote personal growth (Search Institute, 2020). The need for more than “just a relationship” is highlighted in a recent review of mentoring programs that showed nonspecific mentoring approaches had effects that were only half the size of those with more targeted skill-building approaches (Christensen et al., 2020). In sum, adolescents and young adults with foster care experience have a critical need for caring and consistent relationships with adults in addition to structure and skills support that intentionally promotes self-regulation and other developmental competencies. Existing approaches may offer elements of support that are helpful but have clear limitations that likely result in youths’ needs not being fully met. Therefore, exploration of new approaches that more adequately address this group is warranted, particularly from a developmentally informed intervention framework such as co-regulation.

## Co-regulation as a Framework for Promoting Positive Youth Development

Co-regulation is a relatively new framework for thinking about how caring adults can support older youth through relationships, supportive environments, and intentional interactions that build youth self-regulation (Murray et al., 2019). Co-regulation is consistent with the concept of “developmental relationships,” which are characterized by strong emotional connections, responsivity, scaffolding skills at the youth’s level of competency, and shifting power to the youth over time (Li & Julian, 2012). Developmental relationships have also been defined in terms of expressing care, providing support, challenging growth, sharing power, and expanding opportunities (Search Institute, 2020). However, developmental relationships alone provide limited attention to the environment, which is critically important for older youth whose living situations are characterized by instability. Supportive environments provide physical and emotional safety as well as consistency in relationships with caring adults. And even when youths’ home environments change, intentional day-to-day interactions with adults that promote self-reflection, perspective-taking, and learning from experience can be helpful. These types of supportive environments and interactions (the two other key components of co-regulation beyond relationships) help prepare youth for the transition to adulthood not only by building their skills, but also by broadening their perspective, strengthening positive self-beliefs, and building connections with people and resources that facilitate their future goals.

The co-regulation framework adds value to the literature in several ways. First and foremost, it makes youths’ needs for “more than relationships” explicit. Safe, supportive environments and intentional day-to-day interactions to promote youth skills and competencies are also critical for positive youth development. However, they are rarely included in interventions for older youth and young adults (Murray et al., 2016). Indeed, in our team’s review of 59 prevention programs addressing self-regulation for youth aged 14 to 24, none specifically promoted a change in how caring adults interact with youth.

A co-regulation framework also links current developmental science to intervention strategies that can be utilized by caring adults across contexts. Developmental science shows that adolescents and young adults not only have many of the cognitive abilities necessary for self-regulation but also experience intense emotions and are highly sensitive to both stress and peer influence (Kelley et al., 2004; Steinberg, 2014). These processes may reduce their inhibitory control in specific situations and disrupt their abilities to translate knowledge and good intentions into positive actions. Co-regulation considers these specific needs to identify ways caring adults can provide scaffolding through environmental structures (e.g., clear expectations, rules, and boundaries), modeling, and rewards. This allows adults to meet youth where they are developmentally, which leverages youth strengths and provides opportunities for growth while minimizing the potential for risky behaviors. Aligning developmental theory (i.e., self-regulation) and the components of an intervention approach (i.e., co-regulation) also appears likely to support the effectiveness of that approach (e.g., Gottfredson et al., 2015).

A co-regulation framework also broadens the perspective on the nature of youth’s needs, whose behaviors are often attributed to factors internal to the youth, reflecting a bias that overlooks the powerful social ecology (Haydon & Kendall-Taylor, 2016). Although biology and temperament are certainly important, co-regulation shifts the focus to all the ways in which caring adults and the environment also influence development. A co-regulation model also highlights the developmental nature of self-regulation with an opportunity to shift outcomes for youth in meaningful ways through young adulthood and beyond (Murray et al., 2019). Finally, because co-regulation reflects a mindset or approach rather than a set of contextually specific strategies, it can be used by child welfare professionals, mentors, foster care parents, and other caring adults who interact with youth across foster care settings.

## The Present Paper

Given the developmental needs and challenges experienced by older youth (aged 14–24) in the context of foster care, this paper aims to advance the understanding of co-regulation for this population. Additionally, this work was motivated by the interests of a large convening of experts in the child welfare field, including those with lived experience (McKenzie, 2020). This group indicated that co-regulation was highly relevant to older youth with foster care experience near the age of transition to independent living and expressed strong interest in identifying specific co-regulation actions and approaches that could be utilized to better support these youth. In particular, experts noted that focusing on co-regulation highlights opportunities for small but meaningful changes in day-to-day interactions with caring adults. Given the potential for informing practice in meaningful ways, we took an approach known as active engagement, where experts were involved at multiple stages throughout the work (Meyer et al., 2022).

**Fig. 1:**
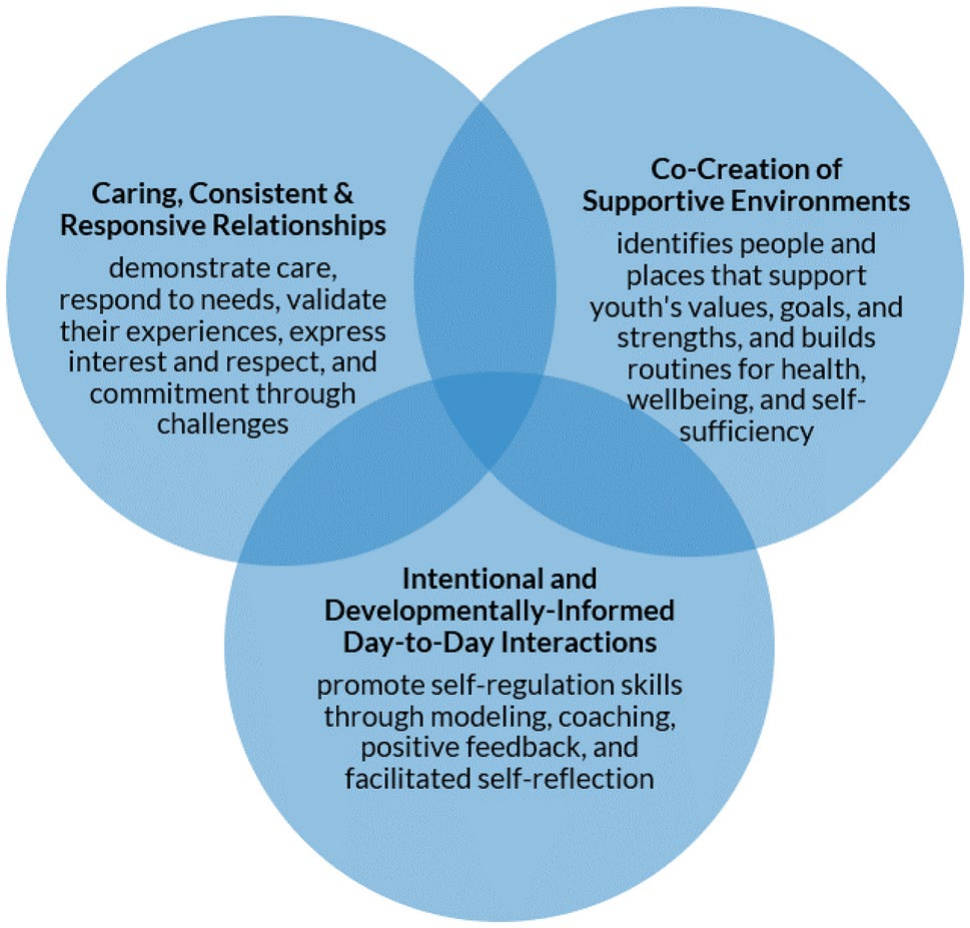
Co-regulation domains

We utilized the *applied contextual model of self-regulation* (Murray et al., 2019), which defines co-regulation as the supportive process by which caring adults and peers promote positive youth development. This definition is a general one that is intended to be modified by age and context. In other words, it takes into account the developmental changes in what self-regulation looks like across ages as well as changes in exactly how co-regulation is enacted with different aged youth in different contexts. For this project focused on older youth in foster care, we further specified our definition based on theoretical and contextual considerations to highlight the importance of consistency and to emphasize older adolescents’ and young adults’ more active roles in co-creating supportive environments. As such, we specifically examined the following: (1) caring, consistent, and responsive relationships; (2) the co-creation of supportive environments; and (3) developmentally informed day-to-day interactions (see Fig. 1). We also focused on youth self-regulation, given its developmental importance and because it is the translational mechanism by which co-regulation is presumed to support social-emotional and behavioral competencies.

We address our aims with a scoping review, which is useful for describing key concepts and characteristics in emerging areas of research, such as co-regulation for youth, where research studies are limited (Sucharew & Macaluso, 2019). Our research questions were as follows: (1) What developmental skills and competencies are addressed in the literature on older youth with foster care experiences in relation to co-regulation? (2) What co-regulation domains and approaches are being used to understand and support this population? and (3) In what contexts are co-regulation supports provided and by whom? By addressing these questions, we hope to move co-regulation theory to practice for this population of interest. Secondarily, we hope to advance understanding of co-regulation in a manner that supports further research with older youth more broadly.

## Method

We followed Arksey and O’Malley’s (2005) methodological framework: (1) Identify the research question; (2) identify relevant articles through a systematic search strategy; (3) select articles based on inclusion and exclusion criteria; (4) chart the data according to key themes; and (5) collate, summarize, and report the results. We also obtained consultation from a team of experts to gain insights that inform and validate findings, which is an optional but highly recommended component of the scoping review process.

### Literature Search Strategies

We utilized the *applied contextual model of self-regulation* (Murray et al., 2019) to develop initial search terms and inclusion/exclusion criteria (see Online Resource 1). This was supplemented with terms recommended by our expert consultants (described below) as well as a review of programs in evidence-based clearinghouses of programs relevant to child welfare, such as the California Evidence-Based Clearinghouse for Child Welfare. We searched for relevant literature in Scopus, ERIC, PsycINFO, and Cochrane databases in April 2020 with no limitations by article publication date or language.

### Article Selection

The title and abstract review were completed by the second author (HR) and a trained research assistant to screen for inclusion/exclusion criteria. Then, two authors (HR, KS) independently reviewed 163 full-text articles to determine eligibility. Any conflicts were resolved through consensus discussion with the first author and content expert (DWM). Online Resource 2 specifies inclusion criteria: (1) peer-reviewed publication or gray literature, (2) empirical study, (3) focused on youth aged 14–24 or the adults who work with them, (4) focused on youth involved in the foster care system or studies that occur in foster placement, kinship care, congregate care, residential care setting, extended services, or aftercare, (5) reflects a co-regulation action and/or capacity, and (6) examines self-regulation or a related skill. Citation tracking was conducted using the Covidence review software (Veritas Health Innovation, 2021).

### Data Extraction and Coding

The research team developed a coding scheme to define co-regulation domains and related constructs, approaches to co-regulation, youth skills and competencies, youth self-regulation skills, co-regulator roles, and special populations (see Online Resource 3). Study design was assessed using a framework for research and evaluation developed by the Administration for Children and Families (OPRE, 2016). To develop the codes, the first, second, and fifth authors (DWM, HR, KS) independently coded five articles, assessed reliability, and operationally defined each code. Through this iterative process, we refined our definition of *approaches to co-regulation* as the manner in which co-regulation was provided (not defined by a specific program or setting).

### Charting and Analysis

To address RQ1, we examined the frequency of youth self-regulation skills across studies. Then, we examined broader competencies and risks in relation to co-regulation, cross-tabulated with self-regulation skills. To address RQ2, we identified the frequency and co-occurrence of the three core co-regulation domains: *relationships*, *day-to-day interactions*, and *supportive environments*. We then charted co-regulation approaches by domains and related constructs. Finally, to address RQ3, we tabulated frequencies for each co-regulator role and qualitatively evaluated the contexts in which each role functioned.

Within our coding scheme, *relationships* are defined by caring, consistency, and responsivity and include recognizing and responding to cues that signal needs and preferences, as well as providing support in times of stress. *Day-to-day interactions* promote self-regulation skills and positive youth development through modeling, prompting, positive feedback, and facilitating self-reflection. *Supportive environments* involve collaborating with youth to ensure their environment is physically and emotionally safe and include people and places that support the youth’s values and goals, work that matches their strengths, and routines that promote health, well-being, and self-sufficiency.

### Active Engagement with Expert Consultants to Facilitate Interpretation

As noted, we consulted with a diverse group of experts, including individuals with lived experience in foster care, therapists and child welfare program directors/staff, federal program staff, and researchers with related expertise, on several occasions over 2 years. This group initially shared perspectives that informed our specification of co-regulation in this population and helped us frame research questions in a way that would have useful practice implications. We also obtained input at other key stages, including identification of search terms. Finally, we met with them for 5 h across 2 days to share perspectives on our findings and identify the next steps for advancing research and creating practice change in the field. Their insights are integrated into our interpretation of findings.

## Results

**Fig. 2:**
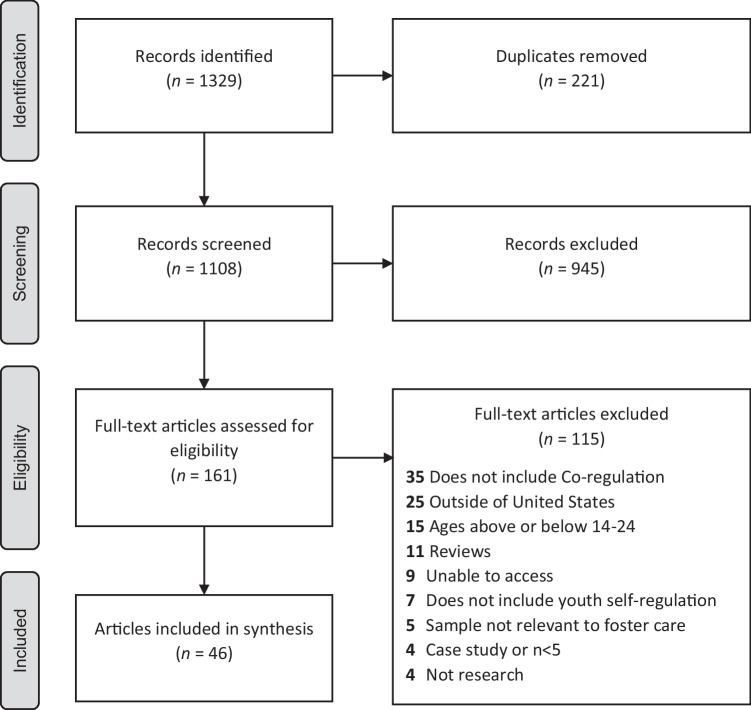
Study flow diagram

The initial search step yielded 1108 unique citations (see Fig. 2). Title and abstract review resulted in 163 full-text articles, with 46 meeting full eligibility criteria. Of those excluded, approximately 30% did not include co-regulation, 22% were conducted outside of the USA, and 13% were not in the eligible age range. See Online Resource 4 for a list of included articles and Online Resource 5 for key codes applied for each.

Approximately two-thirds of the 46 articles (*n* = 30) were descriptive, either foundational (*n* = 8) or exploratory (*n* = 22). Sixteen articles (35%) described program evaluations, a majority at the design and development stage (*n* = 11), with only pilot or implementation data. Two studies were efficacy trials, and three were effectiveness studies. Only three articles were conducted earlier than 2000; nearly half (*n* = 21) were conducted since 2015.

### Developmental Skills, Competencies, and Risks

Online Resource 6 depicts the number of co-regulation articles that addressed developmental competencies and risks in relation to self-regulation skills. In addition to self-regulation, the most common competencies addressed were *educational success* (16 articles; 35%) and *identity development* (13 articles; 28%)*.*

Given the importance of understanding how co-regulation supports self-regulation, we examined 13 different self-regulation and related skills. The most frequently coded was *future orientation* (*n* = 18, 38%); other relatively common skills addressed were *identity-based motivation* (*n* = 12, 26%) and *emotion regulation* (*n* = 10, 21%).

### Co-regulation Domains and Approaches

Online Resource 7 shows the number of articles that addressed each of the core co-regulation domains and their co-occurrence within articles. The relationship domain is addressed in almost all the articles (89%), but only five (11%) included all three domains of co-regulation. Supportive environments and day-to-day interactions were much less common (39% and 28%, respectively). Nearly half of the articles (*n* = 21, 46%) included at least two of the three domains. A handful of articles (primarily in congregate care settings) addressed supportive environments or day-to-day interactions without either of the other two domains, which is not considered ideal according to our model.

Table 1 summarizes eight specific approaches (i.e., the way co-regulation was provided) and how these overlapped with the three co-regulation domains (relationships, supportive environments, and day-to-day interactions) and the two related constructs (peer-co-regulation and self-regulation for the co-regulator). As can be seen, *intentional adult relationships* (where youth are explicitly supported) is the most common approach, although a wide range of approaches address all aspects of co-regulation, albeit with notably fewer approaches and studies addressing co-regulator self-regulation.

**Table 1:** Youth support approaches

Youth support approaches	# of articles	Co-regulation domains and related constructs					Exemplar practices
		Relationships	Environments	Interactions	Peer-co-reg	Co-regulator self-reg	
Intentional adult relationship	20	⬤	●	●	●		Express genuine interest; stay engaged despite challenges; accept youth for who they are but also focus on potential and believe in them; be trustworthy, reliable, and responsive; connect youth to resources and navigate systems
Support from individuals with lived experience	12	⬤	•	•	●		Offer understanding through shared experiences; model success or persistence in the face of adversity
Near-age peer support	10	⬤	•	•	●		Provide a sense of belonging; offer understanding and acceptance; model successful future pathways and how to navigate different challenges
Caregiver training*	8	●	●	•		•	Respond to youth needs with flexibility and support; establish expectations and routines for safety and security; provide guidance for the future; use de-escalation strategies grounded in a strong understanding of youth development
Youth skills support	8	●	•	●	•	•	Use behavior modeling; provide in-the-moment feedback and problem-solving support; help youth apply skills to new challenges; teach skills rather than do it for them; offer patience when youth make mistakes
Cultivating positive self-narrative	7	●	•	•	●		Help youth recognize assets; help youth gain perspective on their experiences; reframe adversity as building resilience
Environmental systems	6	•	●	•	•	•	Work with youth to establish routines and environments that support their goals; encourage them to monitor goal progress
Behavior management	3	•	•	•		•	Provide clear expectations for behavior; provide positive reinforcement; support youth in self-reflection and problem-solving when behavior does not meet expectations; focus on teaching vs. punishing

Based on 35 articles with codable approaches. Multiple approaches may be identified in one article. Examples should not be considered best practices as evidence of efficacy could not be systematically evaluated

• 1 to 4 articles; ● 5 to 9 articles; ⬤ 10 or more articles

^*^Only 2 of these articles focused on parents; the others focused on staff in congregate care facilities

In addition to *intentional adult relationships* (*n* = 20; 57%), other common approaches addressing the relationship domain were *support from individuals with lived experience* (*n* = 12; 34%) and *near-age peer support* (*n* = 10; 29%). Specific approaches that address supportive environments (*n* > 5) were *parent/caregiver training* and *environmental systems*. Day-to-day interactions were most often addressed (*n* ≥ 5) through *youth skills support*. Peer co-regulation was addressed (*n* ≥ 5) in articles with *intentional adult relationships*, *support from individuals with lived experience*, and *near-age peer support*, as well as *cultivating a positive self-narrative*.

*Caregiver training* (*n* = 8; 23%) was used most often to promote relationships and supportive environments. Although a few articles addressed foster parents and kinship caregivers, the majority focused on caregivers in congregate settings. Training focused on understanding youth’s developmental needs and positive discipline practices such as establishing expectations and monitoring youth behavior. *Youth skills support* (*n* = 8, 23%) was provided in programs addressing independent living skills, reproductive health, and employment, among others. *Cultivating a positive self-narrative* (*n* = 7, 20%) was an approach common to educational success and independent living skills programs, often focusing on helping youth recognize their assets and reframe their personal narratives (e.g., adversity builds resilience). *Environmental systems* (*n* = 6, 17%) and *behavior management* (*n* = 3, 9%) approaches were the least common, and most were conducted in residential care settings.

### Co-regulation Contexts and Roles

Online Resource 8 shows the wide range of individuals identified as providing co-regulation (or actions consistent with co-regulation), most commonly mentor (*n* = 15, 33%) and foster parent (*n* = 11, 24%). Mentors were identified most often in college settings and independent living skills programs. Formal and informal peers were identified most often in college settings. Other service providers, such as vocational coaches, were found in programmatic settings such as independent living skills and reproductive health curriculum delivery. Other important adults were referenced most often within education settings.

### Explicit Focus on Self-regulation

Among the 18 articles that specifically addressed self-regulation, most were in residential settings and/or involved mental health care (11 of 18; 61%). A total of 39% of articles (7 of 18) included foster parents, and five (11%) included kinship caregivers. Self-regulation was addressed much less in other settings and for other types of roles, suggesting a way in which future co-regulation research could be enhanced**.**

### Co-regulation Gaps

Several gaps were also identified in the co-regulation literature. Only three of 46 articles (7%) addressed youth of color, and three (7%) specifically addressed youth who are parents. Just one article addressed youth identifying as lesbian, gay, bisexual, transgender, or queer (LGBTQ), and none addressed youth with disabilities. Only seven (15%) articles addressed trauma in a manner that informed co-regulation needs or approaches, despite the fact that many articles referenced experiences of adversity as background or context.

## Discussion

Overall, a co-regulation framework appears to have added value for informing approaches to support the self-regulation development of older youth with foster care experience. It integrates diverse literature into a comprehensive framework that extends beyond a focus on developmental relationships. It links intervention strategies to current developmental science and broadens perspectives on the nature of youth’s needs to the important role played by the child welfare environment. It also suggests ways that caring adults can promote growth in older youth with traumatic histories within challenging circumstances. And while there is not yet specific evidence of how co-regulation might prevent negative outcomes for youth in foster care, this framework is built upon decades of positive parenting research and research demonstrating the value of targeting self-regulation in interventions and building nurturing environments (Murray et al., 2016; Shonkoff et al., 2012).

The literature reviewed indicates that co-regulation is a relevant construct for promoting positive development for older youth and young adults with foster care experience. We found clear examples of each theoretical co-regulation domain and numerous approaches that were being utilized in relation to a wide range of developmental competencies, studied in different contexts and settings, and provided by individuals in many different roles. However, there are several areas where the application of co-regulation may be strengthened, as will be discussed.

### Key Findings

With regard to specific self-regulation skills addressed with co-regulation, future orientation was the most common focus, which is developmentally relevant. However, there was little attention to other self-regulation needs that we would consider critical such as stress management and self-reflection. Co-regulation was also addressed quite often in the context of educational success and identity development, which again seem salient for older adolescents and young adults. Common approaches to co-regulation included mentoring and training for foster care parents, although there was application within a wide range of contexts, including youth development programs, mental health care, and residential settings. However, the application was less frequent with child welfare workers and with teachers, coaches, and other caring adults, insofar as use is reflected in the literature we reviewed.

### Limitations

Published literature on co-regulation is emerging, and what is known is primarily based on qualitative work. This limits the conclusions we can draw regarding associations among constructs, and we are unable to address the effectiveness of co-regulation approaches. Another limitation is that the articles we identified were based on specific inclusion criteria related to our research questions, which may have excluded some relevant work, including programs that focus only on skills instruction for youth. Moreover, we utilized a specific framework to define, code, and interpret co-regulation constructs; using a different framework could reach a different conclusion. We also note that given the rapid growth in publications in this area, there may be new literature in the last few years that was not captured in this study. Finally, we cannot address the extent to which our findings may apply to older youth and young adults beyond those who have foster care experience in the US child welfare system, which was the focus of this review. Despite these limitations, there are several important implications for practice and research.

### Practice Considerations

Although the current literature does not allow for a systematic evaluation of the effects of co-regulation approaches, co-regulation appears to have wide-ranging applications and perceived relevance validated by feedback from our expert consultants. First and most important is that caring adults and older foster care peers in many different roles and types of foster care (e.g., family homes, group homes, residential care facilities) can provide co-regulation support. This may be a mindset shift for some who may perceive trauma and emotion regulation challenges as the purview of trained therapists or professionals. Although professional interventions are certainly important, there are numerous opportunities for caring adults and near-aged peers to promote self-regulation, including educators, sports coaches, employers, and youth program staff, as well as child welfare professionals, foster parents, and kinship caregivers. This aligns well with calls for prevention scientists to adopt broader approaches for promoting well-being beyond programs and specific interventions, particularly by building nurturing environments (Biglan, 2018).

In practice, a co-regulation framework can help caring adults be more intentional in their interactions with youth in a way that promotes self-regulation, regardless of the youth’s history. For example, foster parents who have an older youth placed with them would not only commit to building a connection despite challenging behaviors and provide developmentally appropriate expectations, but they would also help youth recognize their assets and gain perspective on challenging experiences, model emotion regulation skills in the face of stress, facilitate youth self-reflection and problem-solving difficulties, and connect youth to resources that support their interests and future goals. In a group home or residential setting, not only would environments be physically and emotionally safe, but staff would understand how their day-to-day interactions influence youth and would approach them from a strength-based, developmental, and trauma-informed lens. Behavior systems in these settings would build on supportive adult-youth relationships, include plans for co-creating supportive environments, and emphasize day-to-day interactions that support youth in working towards their goals and learning from their mistakes.

Many caring adults already engage in practices that are consistent with co-regulation; the framework can make those actions more explicit and intentional. However, building more supportive environments within the child welfare system will not only require integrating co-regulation principles into training for professionals as well as other caring adults, but also large-scale changes that ensure placement stability and enhance youth’s sense of agency and abilities to navigate the system. Recent federal guidance has taken steps in this direction by promoting normalcy (AECF, 2015) and through efforts to integrate trauma-informed care. Based on feedback from the experts we engaged, it may be helpful to start new intervention efforts by focusing on the well-being of the child welfare workforce, specifically caseworkers, so that they have a greater ability to support youth. Another promising direction is to expand mentoring programs to more intentionally focus on stress management and skills that build long-term resilience, involving near-peer mentors.

Although specific co-regulation strategies may look different in different settings (e.g., homes, classrooms, youth programs), there are specific examples of how this can be applied. In a recent pilot project, a co-regulation framework was utilized to train facilitators working with older youth in the context of relationship education programs, with evidence of feasibility and perceived benefits (Baumgartner et al., 2020). Facilitators reported a shift in mindset regarding their role and approach to youth and began to integrate co-regulation strategies into their interactions in meaningful ways that they described as enhancing youth engagement in programming. To support application across settings, we developed a series of tip sheets that depict scenarios for what co-regulation looks like in different situations for foster parents, kin carers, caseworkers, and teachers interacting with older youth (Murray et al., 2021).

### Research Gaps and Next Steps

Future research on co-regulation should more systematically review programs and practice resources where we found useful approaches not addressed in the empirical literature, such as how individuals with lived experience in foster care, especially near-aged peers, could support youth. Such an approach may contribute to a more robust understanding of co-regulation in this context. To support such practice-based research, the terminology around co-regulation-related constructs should be clarified, for which the present work could serve as a resource.

Although the current work provides valuable perspective on the application of co-regulation for an important population, it is paired with many notable gaps, including (1) a relative lack of approaches that promote supportive environments and day-to-day interactions beyond creating relationships with youth, (2) limited application to employment and career readiness, healthy relationships, and parenting, all of which are important for youth transitioning to adulthood, (3) few studies addressing adult self-regulation skills in order to promote their capacity to provide co-regulation, and (4) lack of literature that meaningfully addresses the co-regulation needs and approaches for youth from racially and ethnically diverse backgrounds as well as those who identify as LGBTQ, who are parenting, or who have disabilities. This fourth gap area was identified as particularly important by our expert consultants and is one where active engagement with youth and other key stakeholders is needed. It is recommended that these gap areas be addressed within the context of practice with stakeholder input, including youth voice.

In order to strengthen confidence in future co-regulation research, greater methodological rigor will be needed, moving beyond descriptive and exploratory studies to evaluate the efficacy of specific strategies and approaches and studying how specific co-regulation factors may predict future well-being and resilience. Such work could provide insight into mechanisms by which co-regulation may serve as an ecological protective factor, such as through the reduction of youth’s physiological stress reactivity, which could further inform program development and practice guidelines. Finally, future research in this area needs practical, reliable measures of co-regulation, perhaps building from measures of parental support (e.g., Cohodes et al., 2021).

Portions of this work have been previously reported in Murray et al. (2021). *Building co-regulation capacity to support positive development for youth with foster care experienc*e, OPRE Report #2021–129, Washington, DC: Office of Planning, Research, and Evaluation, Administration for Children and Families, US Department of Health and Human Services.

## Supplementary Information

Below is the link to the electronic supplementary material.

Supplementary file1 (DOCX 16 KB)

Supplementary file2 (DOCX 20 KB)

Supplementary file3 (DOCX 20 KB)

Supplementary file4 (DOCX 20 KB)

Supplementary file5 (PDF 76 KB)

Supplementary file6 (DOCX 16 KB)

Supplementary file7 (DOCX 16 KB)

Supplementary file8 (TIF 708 KB)

## Data Availability

Available from the first author.
