# Supplementary material for: Co-Regulation as a Support for Older Youth in the Context of Foster Care: a Scoping Review of the Literature
Source: Prev Sci. 2023 Apr 21;24(6):1187–97. doi: 10.1007/s11121-023-01531-3 (PMC10423703; doi:10.1007/s11121-023-01531-3)
Supplement: Supplementary file 1 — Supplementary file1 (DOCX 16 KB) [file 11121_2023_1531_MOESM1_ESM.docx]

**Online Supplemental Resource 1: Scopus Search Terms**

| Search | Query | Items Found |
| --- | --- | --- |
| #1 | TITLE-ABS-KEY ( adolesc* OR youth OR “young adult” OR “transition age”) | 2,855,670 |
| #2 | TITLE-ABS-KEY ( "foster care" OR "foster home" OR "foster family" OR "foster parent" OR "kinship care" OR "kin care" OR "out of home care" OR "out of home placement" OR "relative care" OR "family placement" OR "emancipation" OR "aging out of care" OR "independent living services" OR "foster system" OR "group home" OR "residential care") | 38,179 |
| #3 | TITLE-ABS-KEY ("co-regulat*" OR "transactional regulat*" OR "positive parenting" OR "positive discipline" OR "bond" OR "reciprocal relationship" OR "supportive relationship" OR "warm relationship" OR "responsive relationship" OR "developmental relationship" OR "positive relationship" OR "positive regard" OR "warm regard" OR "cohesive relationship" OR mindful* OR empath* OR acceptance OR "student engagement" OR "collective socialization" OR "belonging" OR "peer support" OR praise OR encourage* OR affirm* OR "social support" OR "emotional support" OR routines OR structure OR "clear expectations" OR "safe risks" OR "class norms" OR "co-creat*" OR scaffold* OR prompt* OR rehears* OR model* OR coach* OR mentor* OR "anticipate problems" OR feedback OR reflect*) | 22,004,117 |
| #4 | TITLE-ABS-KEY  ("self-regulat*" OR "emotion* regulat*" OR "behavior* regulat*" OR coping OR identity OR "possible selves" OR "self-efficacy" OR "self-awareness" OR "self evaluat*" OR "perspective taking" OR "executive function*" OR "problem solv*" OR "decision making" OR "future orient*" OR "goal setting" OR "goal orient*" OR "learning orient*" OR "stress management" OR "sustained engagement" OR "goal commitment" OR persistence OR “effortful” OR “self control” OR “calm down” OR resilien*) | 2,204,328 |
| #5 | #1 AND #2 AND #3 AND #4  TITLE-ABS-KEY ( adolesc* OR youth OR “young adult” OR “transition age”)  AND TITLE-ABS-KEY ( "foster care" OR "foster home" OR "foster family" OR "foster parent" OR "kinship care" OR "kin care" OR "out of home care" OR "out of home placement" OR "relative care" OR "family placement" OR "emancipation" OR "aging out of care" OR "independent living services" OR "foster system" OR "group home" OR "residential care")  AND TITLE-ABS-KEY ("co-regulat*" OR "transactional regulat*" OR "positive parenting" OR "positive discipline" OR "bond" OR "reciprocal relationship" OR "supportive relationship" OR "warm relationship" OR "responsive relationship" OR "developmental relationship" OR "positive relationship" OR "positive regard" OR "warm regard" OR "cohesive relationship" OR mindful* OR empath* OR acceptance OR "student engagement" OR "collective socialization" OR "belonging" OR "peer support" OR praise OR encourage* OR affirm* OR "social support" OR "emotional support" OR routines OR structure OR "clear expectations" OR "safe risks" OR "class norms" OR "co-creat*" OR scaffold* OR prompt* OR rehears* OR model* OR coach* OR mentor* OR "anticipate problems" OR feedback OR reflect*)  AND TITLE-ABS-KEY ("self-regulat*" OR "emotion* regulat*" OR "behavior* regula*" OR coping OR identity OR "possible selves" OR "self-efficacy" OR "self-awareness" OR "self evaluat*" OR "perspective taking" OR "executive function*" OR "problem solv*" OR "decision making" OR "future orient*" OR "goal setting" OR "goal orient*" OR "learning orient*" OR "stress management" OR "sustained engagement" OR "goal commitment" OR persistence OR “effortful” OR “self control” OR “calm down” OR resilien*) | 474 |

Note. #1 = Population, #2 = Setting, #3= Coregulation, #4 = Youth Self-Regulation
