# Supplementary material for: Co-Regulation as a Support for Older Youth in the Context of Foster Care: a Scoping Review of the Literature
Source: Prev Sci. 2023 Apr 21;24(6):1187–97. doi: 10.1007/s11121-023-01531-3 (PMC10423703; doi:10.1007/s11121-023-01531-3)
Supplement: Supplementary file 2 — Supplementary file2 (DOCX 20 KB) [file 11121_2023_1531_MOESM2_ESM.docx]

**Online Resource 1**

*Inclusion/Exclusion Criteria*

| **Criteria** | **Include:** | **Exclude:** |
| --- | --- | --- |
| Publication type | - Peer-reviewed journals; - Gray literature including government reports, program reports and evaluations, and issue papers; | - Books; - Unpublished dissertations; |
| Study design | - Quantitative evaluation (observational, experimental/RCTs, quasi-experimental, longitudinal, meta-analyses); - Qualitative studies; | - Reviews; - Case studies with n<5; |
| Types of participants:  age | - Adolescent to young adults   (14-24 years based on mean for sample);   - Or adults that work with these ages; | - Below or above this age range; |
| Context:  foster Care related | Relevant to youth or young adults who have been in the U.S. foster care system:   - Study occurs in foster placement, kinship care, congregate care, residential care setting, extended services, or after care; - If in restrictive setting such as inpatient care or incarceration, study has a primary population of individuals involved in foster care system; | - Occurs outside of the U.S.; - Study sample may include participants who are involved with foster care system, but they are not specifically discussed; |
| Concept: Co-regulation actions and/or capacity | Examines at least one co-regulation related domain:   - caring, consistent, and responsive relationships; - co-creating the environment; - modeling, prompting, and providing feedback to facilitate development of self-regulation skills; - peer co-regulation; - co-regulator self-regulation; | - Does not meet these criteria; |
| Concept: youth self-regulation | Examines self-regulation OR a related skill (identified by consensus)   - Self-regulation skills: Decision making, Emotion regulation, Future orientation, Identity-based motivation, Persistence, Perspective-taking, Planning, Problem solving, Self-reflection, Stress management - Related skills: Self-determination, Behavior Regulation, Resilience | - Does not meet these criteria. |
