# Supplementary material for: Co-Regulation as a Support for Older Youth in the Context of Foster Care: a Scoping Review of the Literature
Source: Prev Sci. 2023 Apr 21;24(6):1187–97. doi: 10.1007/s11121-023-01531-3 (PMC10423703; doi:10.1007/s11121-023-01531-3)
Supplement: Supplementary file 3 — Supplementary file3 (DOCX 20 KB) [file 11121_2023_1531_MOESM3_ESM.docx]

**Online Resource 3. Coding Scheme**

**Study Design**

- Foundational descriptive
- Exploratory descriptive
- Design and development
- Efficacy
- Effectiveness
- Scale-Up

**Co-regulation Domains**

- Caring, Consistent, & Responsive Relationships
- Co-Creation of Supportive Environments
- Intentional and Developmentally-Informed Day-to-Day Interactions

**Other Co-regulation Related Constructs**

- Peer co-regulation
- Co-regulator self-regulation or capacity to provide co-regulation

**Approaches**

- Intentional adult relationship
- Near-age peer support
- Support from individual with lived experience
- Youth skills support (coaching)
- Environmental systems or supports
- Behavioral management
- Cultivating positive self-narrative
- Parent/caregiver training
- None

**Youth Skills and Competencies**

- College success
- Employment and career planning
- Healthy relationships
- Identity development
- Increased social capital
- Independent living skills
- Decreased sexual risk behavior
- Decreased substance use
- Self-regulation

**Youth Self-regulation Skills**

- Decision making
- Emotion regulation
- Future orientation
- Identity-based motivation
- Persistence
- Perspective-taking
- Planning
- Problem solving
- Self-reflection
- Stress management
- Self-determination
- Behavior regulation
- Resilience

**Co-regulator Roles**^^[[1]](#footnote-1)^^

- Parent
- Child welfare service provider
- Employer
- Extracurricular advisor (e.g.., coach)
- Formal peer
- Foster parent
- Informal peer
- Kinship caregiver
- Mental health service provider
- Mentor
- Other family members
- Other important adults
- Other service provider
- Residential staff/caregiver
- Sibling
- Teacher

**Focus on Special Populations**

- LGBTQ Youth
- Youth with disabilities
- Youth who are parenting
- Youth of color

1. Parent refers to youth’s birth parents or family of origin, whereas foster parent refers to non-related caregivers in family foster care settings. [↑](#footnote-ref-1)
