# Supplementary material for: Co-Regulation as a Support for Older Youth in the Context of Foster Care: a Scoping Review of the Literature
Source: Prev Sci. 2023 Apr 21;24(6):1187–97. doi: 10.1007/s11121-023-01531-3 (PMC10423703; doi:10.1007/s11121-023-01531-3)
Supplement: Supplementary file 4 — Supplementary file4 (DOCX 20 KB) [file 11121_2023_1531_MOESM4_ESM.docx]

**Online Resource 5**

*Included Articles*

Ahrens, K. R., Spencer, R., Bonnar, M., Coatney, A., & Hall, T. (2016). Qualitative evaluation of historical and relational factors influencing pregnancy and sexually transmitted infection risks in foster youth. Children and Youth Services Review, 61(PG-245-252), 245–252. https://doi.org/10.1016/j.childyouth.2015.12.027

Albertson, K., Crouch, J. M., Udell, W., Schimmel-Bristow, A., Serrano, J., & Ahrens, K. R. (2020). Caregiver-endorsed strategies to improving sexual health outcomes among foster youth. Child and Family Social Work. https://doi.org/10.1111/cfs.12726

Augsberger, A., Springwater, J. S., Hilliard-Koshinsky, G., Barber, K., & Martinez, L. S. (2019). Youth participation in policy advocacy: Examination of a multi-state former and current foster care youth coalition. Children and Youth Services Review, 107. https://doi.org/10.1016/j.childyouth.2019.104491

Bermea, A. M., Forenza, B., Rueda, H. A., & Toews, M. L. (2019). Resiliency and Adolescent Motherhood in the Context of Residential Foster Care. Child and Adolescent Social Work Journal, 36(5), 459–470. https://doi.org/10.1007/s10560-018-0574-0

Boel-Studt, S., Schelbe, L., Deichen Hansen, M., & Tobia, L. (2018). Increasing Youth Engagement in Residential Group Care: A Mixed Methods Pilot Study of a Youth-Guided Incentive Program. Child & Youth Care Forum, 47(6), 863–880. http://dx.doi.org/10.1007/s10566-018-9465-y NS -

Bowen, E., Ball, A., Semanchin Jones, A., & Irish, A. (2018). Striving and Dreaming: A Grounded Theory of the Transition to Adulthood for Cross-Systems Youth. Youth and Society. https://doi.org/10.1177/0044118X18791869

Brown, A. D., McCauley, K., Navalta, C. P., & Saxe, G. N. (2013). Trauma Systems Therapy in Residential Settings: Improving Emotion Regulation and the Social Environment of Traumatized Children and Youth in Congregate Care. Journal of Family Violence, 28(7), 693–703. https://doi.org/10.1007/s10896-013-9542-9

Day, A. G., Baroni, B., Somers, C., Shier, J., Zammit, M., Crosby, S., et al. (2017). Trauma and Triggers: Students’ Perspectives on Enhancing the Classroom Experiences at an Alternative Residential Treatment-Based School. Children & Schools, 39(4), 227–237. http://dx.doi.org/10.1093/cs/cdx018 NS -

Dunn, L. T. (2010). Shifting Gears: From Coercion to Respect in Residential Care. Reclaiming Children and Youth, 19(1), 40–44. http://reclaimingjournal.com/issues-7 NS -

Geenen, S., Powers, L. E., Phillips, L. A., Nelson, M., McKenna, J., Winges-Yanez, N., et al. (2015). Better futures: A randomized field test of a model for supporting young people in foster care with mental health challenges to participate in higher education. The Journal of Behavioral Health Services & Research, 42(2), 150–171. https://doi.org/10.1007/s11414-014-9451-6

Geiger, J. M., Cheung, J. R., Hanrahan, J. E., Lietz, C. A., & Carpenter, B. M. (2017). Increasing Competency, Self-Confidence, and Connectedness Among Foster Care Alumni Entering a 4-Year University: Findings from an Early-Start Program. Journal of Social Service Research, 43(5), 566–579. https://doi.org/10.1080/01488376.2017.1342307

Hass, M., & Graydon, K. (2009). Sources of resiliency among successful foster youth. Children and Youth Services Review, 31(4), 457–463. https://doi.org/10.1016/j.childyouth.2008.10.001

Hines, A. M., Merdinger, J., & Wyatt, P. (2005). Former foster youth attending college: Resilience and the transition to young adulthood. American Journal of Orthopsychiatry, 75(3), 381–394. https://doi.org/10.1037/0002-9432.75.3.381

Hudson, A. L. (2013). Career mentoring needs of youths in foster care: Voices for change. Journal of Child and Adolescent Psychiatric Nursing, 26(2), 131–137. https://doi.org/10.1111/jcap.12032

Iglehart, A. P., & Becerra, R. M. (2002). Hispanic and African American Youth: Life after Foster Care Emancipation. Journal of Ethnic & Cultural Diversity in Social Work, 11(1-2), 79–107. https://search.ebscohost.com/login.aspx?direct=true&db=eric&AN=EJ675311&site=ehost-live&scope=site NS -

Johnson, R. M., Strayhorn, T. L., & Parler, B. (2020). “I just want to be a regular kid:” A qualitative study of sense of belonging among high school youth in foster care. Children and Youth Services Review, 111. https://doi.org/10.1016/j.childyouth.2020.104832

Jones, L. (2013). The Family and Social Networks of Recently Discharged Foster Youth. Journal of Family Social Work, 16(3), 225–242. https://doi.org/10.1080/10522158.2013.786307

Jones, M. A., & Williams, M. A. (1983). Collaborative Wellness Counseling with a Group of Black Adolescents. https://search.ebscohost.com/login.aspx?direct=true&db=eric&AN=ED234571&site=ehost-live&scope=site NS -

Kirk, R., & Day, A. (2011). Increasing college access for youth aging out of foster care: Evaluation of a summer camp program for foster youth transitioning from high school to college. Children and Youth Services Review, 33(7), 1173–1180. https://doi.org/10.1016/j.childyouth.2011.02.018

Lovitt, T., & Emerson, J. (2009). Foster Youth Who Have Succeeded in Higher Education: Common Themes. Journal of the American Academy of Special Education Professionals, 18-23. https://search.ebscohost.com/login.aspx?direct=true&db=eric&AN=EJ1137377&site=ehost-live&scope=site NS -

Mallon, G. P., Aledort, N., & Ferrera, M. (2002). There’s No Place Like Home: Achieving Safety, Permanency, and Well-Being for Lesbian and Gay Adolescents in Out-of-Home Care Settings. Child Welfare, 81(2), 407–439. https://search.ebscohost.com/login.aspx?direct=true&db=eric&AN=EJ649367&site=ehost-live&scope=site NS -

Mauzerall, H. A. (1983). Emancipation from foster care: The independent living project. Child Welfare: Journal of Policy, Practice, and Program, 62(1), 46–53. https://search.ebscohost.com/login.aspx?direct=true&db=psyh&AN=1983-28662-001&site=ehost-live&scope=site NS -

McMillen, J. C., Narendorf, S. C., Robinson, D., Havlicek, J., Fedoravicius, N., Bertram, J., & McNelly, D. (2015). Development and piloting of a treatment foster care program for older youth with psychiatric problems. Child and Adolescent Psychiatry and Mental Health, 9(1). https://doi.org/10.1186/s13034-015-0057-4

Neal, D. (2017). Academic resilience and caring adults: The experiences of former foster youth. Children and Youth Services Review, 79, 242-248. https://doi.org/10.1016/j.childyouth.2017.06.005

Nsonwu, M. B., Dennison, S., & Long, J. (2015). Foster Care Chronicles: Use of the Arts for Teens Aging Out of the Foster Care System. Journal of Creativity in Mental Health, 10(1), 18–33. https://doi.org/10.1080/15401383.2014.935546

Opsal, T., & Eman, R. (2018). Invisible vulnerability: Participant perceptions of a campus-based program for students without caregivers. Children and Youth Services Review, 94, 617-627. https://doi.org/10.1016/j.childyouth.2018.09.002

Osterling, K. L., & Hinest, A. M. (2006). Mentoring adolescent foster youth: Promoting resilience during developmental transitions. Child and Family Social Work, 11(3), 242–253. https://doi.org/10.1111/j.1365-2206.2006.00427.x

Patterson, D., Day, A., Vanderwill, L., Willis, T., Resko, S., Henneman, K., & Cohick, S. (2018). Identifying the essential competencies for resource parents to promote permanency and well-being of adolescents in care. Children and Youth Services Review, 88, 457-466. https://doi.org/10.1016/j.childyouth.2018.03.041

Piel, M. H., & Lacasse, J. R. (2017). Responsive engagement in mental health services for foster youth transitioning to adulthood. Journal of Family Social Work, 20(4), 340–356. https://doi.org/10.1080/10522158.2017.1348115

Radey, M., Schelbe, L., McWey, L. M., Holtrop, K., & Canto, A. I. (2016). “It’s really overwhelming”: Parent and service provider perspectives of parents aging out of foster care. Children and Youth Services Review, 67, 1-10. https://doi.org/10.1016/j.childyouth.2016.05.013

Rios, S. J., & Rocco, T. S. (2014). From Foster Care to College: Barriers and Supports on the Road to Postsecondary Education. Emerging Adulthood, 2(3), 227–237. https://doi.org/10.1177/2167696814526715

Rivard, J. C., Bloom, S. L., Abramovitz, R., Pasquale, L. E., Duncan, M., McCorkle, D., & Gelman, A. (2003). Assessing the implementation and effects of a trauma-focused intervention for youths in residential treatment. Psychiatric Quarterly, 74(2), 137–154. https://doi.org/10.1023/A:1021355727114

Rivard, J. C., Bloom, S. L., McCorkle, D., & Abramovitz, R. (2005). Preliminary results of a study examining the implementation and effects of a trauma recovery framework for youths in residential treatment. Therapeutic Communities, 26(1), 79–92. https://www.scopus.com/inward/record.uri?eid=2-s2.0-33745671069&partnerID=40&md5=62acd82bdfb95bec8f4905728bd00890 NS -

Rivard, J. C., McCorkle, D., Duncan, M. E., Pasquale, L. E., Bloom, S. L., & Abramovitz, R. (2004). Implementing a Trauma Recovery Framework for Youths in Residential Treatment. Child & Adolescent Social Work Journal, 21(5), 529–550. https://doi.org/10.1023/B:CASW.0000043363.14978.e6

Rosenwald, M., McGhee, T., & Noftall, R. (2013). Perspectives on Independent Living Services Among Resilient Youth. Journal of Family Social Work, 16(2), 148–163. https://doi.org/10.1080/10522158.2013.765816

Sakai, C., Mackie, T. I., Shetgiri, R., Franzen, S., Partap, A., Flores, G., & Leslie, L. K. (2014). Mental health beliefs and barriers to accessing mental health services in youth aging out of foster care. Academic Pediatrics, 14(6), 565–573. https://doi.org/10.1016/j.acap.2014.07.003

Samuels, G. M., & Pryce, J. M. (2008). “What doesn’t kill you makes you stronger”: Survivalist self-reliance as resilience and risk among young adults aging out of foster care. Children and Youth Services Review, 30(10), 1198–1210. https://doi.org/10.1016/j.childyouth.2008.03.005

Skelton, E. A., Crosland, K. A., & Clark, H. B. (2016). Acquisition of a Social Problem-Solving Method by Caregivers in the Foster Care System: Evaluation and Implications. Child and Family Behavior Therapy, 38(1), 32–46. https://doi.org/10.1080/07317107.2016.1135699

Spencer, R., Gowdy, G., Drew, A. L., & Rhodes, J. E. (2019). “Who Knows Me the Best and Can Encourage Me the Most?”: Matching and Early Relationship Development in Youth-Initiated Mentoring Relationships with System-Involved Youth. Journal of Adolescent Research, 34(1), 3–29. http://dx.doi.org/10.1177/0743558418755686 NS -

Storer, H. L., Barkan, S. E., Stenhouse, L. L., Eichenlaub, C., Mallillin, A., & Haggerty, K. P. (2014). In search of connection: The foster youth and caregiver relationship. Children and Youth Services Review, 42, 110-117. https://doi.org/10.1016/j.childyouth.2014.04.008

Strolin-Goltzman, J., Woodhouse, V., Suter, J., & Werrbach, M. (2016). A mixed method study on educational well-being and resilience among youth in foster care. Children and Youth Services Review, 70, 30-36. https://doi.org/10.1016/j.childyouth.2016.08.014

Taylor, R. J., Shade, K., Lowry, S. J., & Ahrens, K. (2020). Evaluation of reproductive health education in transition-age youth. Children and Youth Services Review, 108. https://doi.org/10.1016/j.childyouth.2019.104530

Uzoebo, V. N., Kioko, M., & Jones, R. (2008). Deconstructing youth transition to adulthood services: Lessons learned from the VISIONS program. Vulnerable Children and Youth Studies, 3(1), 37–41. https://doi.org/10.1080/17450120701767688

Vorhies, V., Davis, K. E., Frounfelker, R. L., & Kaiser, S. M. (2012). Applying social and cultural capital frameworks: Understanding employment perspectives of transition age youth with serious mental health conditions. Journal of Behavioral Health Services and Research, 39(3), 257–270. https://doi.org/10.1007/s11414-012-9274-2

Watt, T. T., Norton, C. L., & Jones, C. (2013). Designing a campus support program for foster care alumni: Preliminary evidence for a strengths framework. Children and Youth Services Review, 35(9), 1408–1417. https://doi.org/10.1016/j.childyouth.2013.06.002

Yancey, A. K. (1998). Building positive self-image in adolescents in foster care: The use of role models in an interactive group approach. Adolescence, 33(130), 253–267. https://www.scopus.com/inward/record.uri?eid=2-s2.0-0347038799&partnerID=40&md5=2fb02310bed34b8eb6e62a165da8958e NS -
