# Supplementary material for: Co-Regulation as a Support for Older Youth in the Context of Foster Care: a Scoping Review of the Literature
Source: Prev Sci. 2023 Apr 21;24(6):1187–97. doi: 10.1007/s11121-023-01531-3 (PMC10423703; doi:10.1007/s11121-023-01531-3)
Supplement: Supplementary file 5 — Supplementary file5 (PDF 76 KB) [file 11121_2023_1531_MOESM5_ESM.pdf]

Study	Domains	Related Contstructs	Approaches	Youth Skills and Competencies	Youth Self-regulation Skills	Co-regulator Roles	Focus on Special Populations
Ahrens, K. R., Spencer, R., Bonnar, M., Coatney, A., & Hall, T. (2016)	Relationships		Support from individuals with lived experience; Parent/caregiver training	Decreased sexual risk behavior; Self-regulation	Emotion regulation; Planning	Foster parent; Kinship caregiver; Other service provider	
Albertson, K., Crouch, J. M., Udell, W., Schimmel-Bristow, A., Serrano, J., & Ahrens, K. R. (2020)	Relationships; Environments		Parent/caregiver training	Decreased sexual risk behavior; Self-regulation	Decision making; Future orientation	Foster parent; Kinship caregiver	
Augsberger, A., Springwater, J. S., Hilliard-Koshinsky, G., Barber, K., & Martinez, L. S. (2019)	Relationships		Intentional adult relationship; Near age peer support; Support from individuals with lived experience	Increased social capital	Decision making; Identity based motivation; Behavioral regulation	Extracurricular advisor (e.g., coach); Formal peer	
Bermea, A. M., Forenza, B., Rueda, H. A., & Toews, M. L. (2019)	Relationships	Peer co-regulation	Near age peer support; Support from individuals with lived experience	Identity development	Emotion regulation; Future orientation; Identity based motivation	Informal peer; Other family members; Residential staff/caregiver	Youth who are parenting
Boel-Studt, S., Schelbe, L., Deichen Hansen, M., & Tobia, L. (2018)	Relationships; Environments		Intentional adult relationship; Behavioral Management	Self-regulation	Resilience	Mental health service provider	
Bowen, E., Ball, A., Semanchin Jones, A., & Irish, A. (2018)	Relationships		Intentional adult relationship	Identity development	Future orientation; Perspective-taking; Planning; Behavioral regulation	Parent; Child welfare service provider; Other family members	
Brown, A. D., McCauley, K., Navalta, C. P., & Saxe, G. N. (2013)	Relationships; Environments		Parent/caregiver training	Self-regulation	Emotion regulation	Parent; Foster parent; Kinship caregiver; Residential staff/caregiver	
Day, A. G., Baroni, B., Somers, C., Shier, J., Zammit, M., Crosby, S., et al. (2017)	Relationships; Environments		Environmental systems or supports	College success; Self-regulation	Emotion regulation; Behavioral regulation	Residential staff/caregiver; Teacher	
Dunn, L. T. (2010)	Relationships; Environments; Interactions	Co-regulator self-regulation	Youth skills support (coaching); Environmental systems or supports; Behavioral Management	Self-regulation	Self-reflection	Residential staff/caregiver	
Geenen, S., Powers, L. E., Phillips, L. A., Nelson, M., McKenna, J., Winges-Yanez, N., et al. (2015)	Relationships; Interactions	Peer co-regulation	Intentional adult relationship; Near age peer support; Support from individuals with lived experience; Youth skills support (coaching); Cultivating positive self-narrative	College success; Identity development; Self-regulation	Future orientation; Identity based motivation; Planning	Formal peer; Mentor	
Geiger, J. M., Cheung, J. R., Hanrahan, J. E., Lietz, C. A., & Carpenter, B. M. (2017)	Relationships	Peer co-regulation	Intentional adult relationship; Near age peer support; Support from individuals with lived experience	College success; Identity development; Increased social capital; Independent living skills; Self-regulation	Persistence	Formal peer; Mentor	

Hass, M., & Graydon, K. (2009)	Relationships	Peer co-regulation	None	College success; Self-regulation	Problem solving	Informal peer; Mentor	
Hines, A. M., Merdinger, J., & Wyatt, P. (2005)	Relationships		Intentional adult relationship	College success; Self-regulation	Future orientation	Foster parent; Kinship caregiver	
Hudson, A. L. (2013)	Relationships		None	College success; Employment and career planning	Future orientation	Mentor	
Iglehart, A. P., & Becerra, R. M. (2002)	Relationships	Peer co-regulation	None	Increased social capital; Independent living skills	Planning	Child welfare service provider; Informal peer	Youth of color
Johnson, R. M., Strayhorn, T. L., & Parler, B. (2020)	Relationships	Peer co-regulation	None	College success; Identity development			
Jones, L. (2013)	Relationships	Peer co-regulation	Intentional adult relationship	None	Behavioral regulation	Parent; Foster parent; Informal peer; Other family members	
Jones, M. A., & Williams, M. A. (1983)	Relationships; Environments; Interactions	Peer co-regulation	Intentional adult relationship; Near age peer support; Support from individuals with lived experience	Self-regulation	Self-reflection	Formal peer; Mental health service provider	Youth of color
Kirk, R., & Day, A. (2011)	Relationships	Peer co-regulation	Near age peer support; Support from individuals with lived experience; Cultivating positive self-narrative; None	College success; Employment and career planning	Future orientation; Persistence; Planning	Formal peer; Mentor	
Lovitt, T., & Emerson, J. (2009)	Relationships		None	College success	Future orientation; Persistence	Foster parent; Other family members	
Mallon, G. P., Aledort, N., & Ferrera, M. (2002)	Relationships	Co-regulator self-regulation	None	Identity development; Independent living skills			LGBTQ youth
Mauzerall, H. A. (1983)	Relationships; Environments	Peer co-regulation	Intentional adult relationship; Near age peer support; Support from individuals with lived experience; Youth skills support (coaching)	Employment and career planning; Independent living skills	Planning; Self-reflection	Child welfare service provider; Formal peer	
McMillen, J. C., Narendorf, S. C., Robinson, D., Havlicek, J., Fedoravicius, N., Bertram, J., & McNelly, D. (2015)	Relationships; Environments; Interactions		Intentional adult relationship; Behavioral Management; Parent/caregiver training	Independent living skills; Self-regulation	Emotion regulation	Foster parent; Mental health service provider; Mentor	
Neal, D. (2017)	Relationships; Environments		Intentional adult relationship	College success	Future orientation; Self-reflection; Resilience	Extracurricular advisor (e.g., coach); Other family members; Other important adults; Teacher	
Nsonwu, M. B., Dennison, S., & Long, J. (2015)	Relationships; Environments	Peer co-regulation	Near age peer support; Support from individuals with lived experience; Cultivating positive self-narrative	Identity development	Self-reflection	Formal peer; Mental health service provider	

Opsal, T., & Eman, R. (2018)	Relationships	Peer co-regulation	Near age peer support; Support from individuals with lived experience	College success; Identity development	Problem solving; Self-determination	Informal peer; Other service provider	
Osterling, K. L., & Hinest, A. M. (2006)	Relationships; Interactions		Intentional adult relationship; Youth skills support (coaching)	College success; Independent living skills	Emotion regulation; Future orientation; Self-determination	Mentor	
Patterson, D., Day, A., Vanderwill, L., Willis, T., Resko, S., Henneman, K., & Cohick, S. (2018)	Relationships; Environments; Interactions	Co-regulator self-regulation	Parent/caregiver training	Identity development; Increased social capital; Self-regulation	Resilience	Foster parent; Kinship caregiver	
Piel, M. H., & Lacasse, J. R. (2017)	Relationships; Interactions		None		Decision making; Self-determination	Informal peer; Mental health service provider; Mentor; Other important adults	
Radey, M., Schelbe, L., McWey, L. M., Holtrop, K., & Canto, A. I. (2016)	Relationships	Peer co-regulation	None		Identity based motivation	Informal peer; Other important adults	Youth who are parenting
Rios, S. J., & Rocco, T. S. (2014)	Relationships; Environments		None	College success	Decision making; Future orientation; Identity based motivation; Persistence; Planning; Problem solving; Self-determination	Child welfare service provider; Foster parent; Mentor; Other family members; Other important adults	
Rivard, J. C., Bloom, S. L., Abramovitz, R., Pasquale, L. E., Duncan, M., McCorkle, D., & Gelman, A. (2003)	Environments		Environmental systems or supports	Self-regulation	Emotion regulation; Future orientation; Problem solving; Behavioral regulation	Mental health service provider; Residential staff/caregiver	
Rivard, J. C., Bloom, S. L., McCorkle, D., & Abramovitz, R. (2005)	Environments		Environmental systems or supports; Parent/caregiver training	Self-regulation	Decision making; Future orientation; Perspective-taking; Problem solving; Stress management	Mental health service provider; Residential staff/caregiver	
Rivard, J. C., McCorkle, D., Duncan, M. E., Pasquale, L. E., Bloom, S. L., & Abramovitz, R. (2004)	Environments		Environmental systems or supports; Parent/caregiver training	Self-regulation	Decision making; Emotion regulation; Future orientation; Stress management; Behavioral regulation	Mental health service provider; Other family members; Residential staff/caregiver	
Rosenwald, M., McGhee, T., & Noftall, R. (2013)	Relationships; Interactions		Intentional adult relationship; Youth skills support (coaching)	Independent living skills	Future orientation; Identity based motivation; Self-reflection; Self-determination	Mentor; Other service provider	Youth who are parenting
Sakai, C., Mackie, T. I., Shetgiri, R., Franzen, S., Partap, A., Flores, G., & Leslie, L. K. (2014)	Relationships		Intentional adult relationship		Emotion regulation; Stress management	Mental health service provider	

Samuels, G. M., & Pryce, J. M. (2008)	Relationships		None	Identity development	Identity based motivation; Self-reflection	Parent; Kinship caregiver	
Skelton, E. A., Crosland, K. A., & Clark, H. B. (2016)	Interactions		Parent/caregiver training	Self-regulation	Decision making; Problem solving; Resilience	Residential staff/caregiver	
Spencer, R., Gowdy, G., Drew, A. L., & Rhodes, J. E. (2019)	Relationships; Interactions		Intentional adult relationship	Increased social capital	Future orientation; Planning; Resilience	Mentor; Other service provider	
Storer, H. L., Barkan, S. E., Stenhouse, L. L., Eichenlaub, C., Mallillin, A., & Haggerty, K. P. (2014)	Relationships; Environments; Interactions		Intentional adult relationship; Cultivating positive self-narrative	College success; Independent living skills; Self-regulation	Future orientation	Foster parent	
Strolin-Goltzman, J., Woodhouse, V., Suter, J., & Werrbach, M. (2016)	Relationships; Environments	Peer co-regulation	Environmental systems or supports; Cultivating positive self-narrative	College success	Future orientation; Identity based motivation; Self-determination	Child welfare service provider; Extracurricular advisor (e.g., coach); Foster parent; Informal peer; Kinship caregiver; Other important adults; Other service provider; Teacher	
Taylor, R. J., Shade, K., Lowry, S. J., & Ahrens, K. (2020)	Environments		Youth skills support (coaching)	Decreased sexual risk behavior	Problem solving	Other service provider	
Uzoebo, V. N., Kioko, M., & Jones, R. (2008)	Relationships; Interactions		Intentional adult relationship; Youth skills support (coaching)	Identity development; Independent living skills	Identity based motivation; Self-determination	Mentor	
Vorhies, V., Davis, K. E., Frounfelker, R. L., & Kaiser, S. M. (2012)	Relationships; Interactions		Intentional adult relationship; Youth skills support (coaching)	Employment and career planning; Increased social capital	Emotion regulation; Identity based motivation; Self-reflection	Employer; Mentor	
Watt, T. T., Norton, C. L., & Jones, C. (2013)	Relationships	Peer co-regulation	Intentional adult relationship; Near age peer support; Support from individuals with lived experience; Cultivating positive self-narrative	College success; Identity development; Increased social capital	Identity based motivation	Informal peer; Mentor	
Yancey, A. K. (1998)	Relationships		Intentional adult relationship; Support from individuals with lived experience; Cultivating positive self-narrative	Identity development	Identity based motivation; Stress management	Mentor	Youth of color
