# Supplementary material for: Co-Regulation as a Support for Older Youth in the Context of Foster Care: a Scoping Review of the Literature
Source: Prev Sci. 2023 Apr 21;24(6):1187–97. doi: 10.1007/s11121-023-01531-3 (PMC10423703; doi:10.1007/s11121-023-01531-3)
Supplement: Supplementary file 6 — Supplementary file6 (DOCX 16 KB) [file 11121_2023_1531_MOESM6_ESM.docx]

| **Online Resource 6**  *Skills, Competencies and Risks Discussed in Relation to Self-Regulation and Co-Regulation Practices* | | |
| --- | --- | --- |
| **Skills, Competencies and Risks** | **Self-regulation** | **Total** |
| Self-regulation | - | **18** |
| Educational success | 6 | **16** |
| Identity development | 3 | **13** |
| Independent living skills | 3 | **9** |
| Increased social capital | 2 | **7** |
| Employment & career readiness | 0 | **4** |
| Decreased sexual risk behavior | 2 | **4** |
| Decreased substance use | 0 | **2** |
| Healthy relationships | 0 | **0** |
| *Note*. Multiple skills, competencies, and risks could be coded per article. | | |
