# Supplementary material for: Co-Regulation as a Support for Older Youth in the Context of Foster Care: a Scoping Review of the Literature
Source: Prev Sci. 2023 Apr 21;24(6):1187–97. doi: 10.1007/s11121-023-01531-3 (PMC10423703; doi:10.1007/s11121-023-01531-3)
Supplement: Supplementary file 7 — Supplementary file7 (DOCX 16 KB) [file 11121_2023_1531_MOESM7_ESM.docx]

| **Online Resource 7**  *Co-Regulator Roles* | |
| --- | --- |
| **Role** | **# of Articles** |
| Mentor | 15 |
| Foster parent | 11 |
| Informal peer | 9 |
| Mental health service provider | 9 |
| Residential staff/caregiver | 8 |
| Formal peer | 7 |
| Kinship caregiver | 7 |
| Other family members | 6 |
| Other service provider | 6 |
| Child welfare service provider | 5 |
| Other important adults | 5 |
| Parent | 4 |
| Extracurricular advisor | 3 |
| Teacher | 3 |
| Employer | 1 |
| Sibling | 0 |
| *Note.* More than one role may be identified per article. | |
