# Supplementary figures and images for: Co-Regulation as a Support for Older Youth in the Context of Foster Care: a Scoping Review of the Literature
Source: Prev Sci. 2023 Apr 21;24(6):1187–97. doi: 10.1007/s11121-023-01531-3 (PMC10423703; doi:10.1007/s11121-023-01531-3)

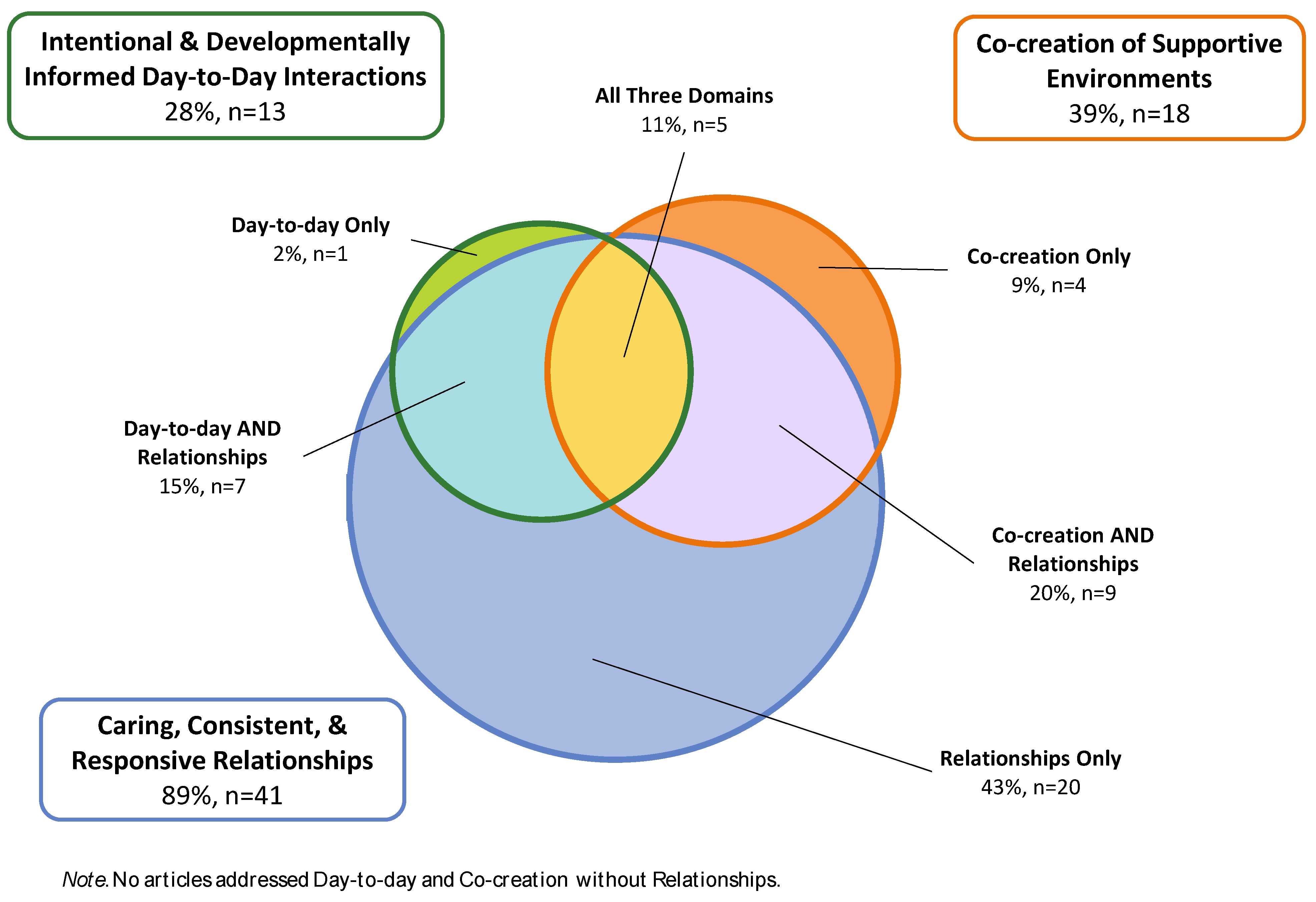

Supplement: Supplementary file 8 — Supplementary file8 (TIF 708 KB) [file 11121_2023_1531_MOESM8_ESM.tif]
